# Nomogram model for predicting papillary thyroid carcinoma based on clinical-ultrasound characteristics and inflammatory biomarkers: a multicenter study

**DOI:** 10.3389/fonc.2026.1752376

**Published:** 2026-04-13

**Authors:** Caihong Wang, Ying Yang, Yuyu Hua, Dan Qin, Yan Cai, Ruheng Feng, Jing Wen

**Affiliations:** 1 Academy of Medical Imaging, Guizhou Medical University, Guiyang, Guizhou, China; 2 The Second People’s Hospital of Guizhou Province, Guiyang, Guizhou, China; 3 Department of Ultrasound Center, Affiliated Hospital of Guizhou Medical University, Guiyang, Guizhou, China

**Keywords:** LMR, NLR, nomogram, papillary thyroid carcinoma, PLR, ultrasound

## Abstract

**Objectives:**

The predictive significance of lymphocyte-related inflammatory biomarkers for papillary thyroid carcinoma (PTC) remains unclear. This study aims to provide a new tool for differentiating PTC from benign thyroid nodule (BTN) by constructing a nomogram model.

**Methods:**

Institution A (n=733) was randomly divided into a training cohort (n=513) and an internal validation cohort (n=220) at a 7:3 ratio. Institution B (n=164) served as external validation cohort 1, while Institutions C and D (n=143) were combined as external validation cohort 2. In the training cohort, a nomogram model was constructed by stepwise selection of features through univariate and multivariate logistic regression. The model’s discrimination, calibration, and clinical applicability were assessed using the area under the receiver operating characteristic curve (AUC), decision curve analysis (DCA), and clinical impact curve (CIC).

**Results:**

The final nomogram integrated the inflammatory biomarker NLR with patient age and Ultrasound(US) features. This model demonstrated excellent predictive performance across the training cohort (AUC 0.841, 95%CI: 0.807-0.872), internal validation cohort (AUC 0.828, 95%CI: 0.772-0.876), external validation cohort 1 (AUC 0.756, 95%CI: 0.683-0.820), and external validation cohort 2 (AUC 0.833, 95%CI: 0.762-0.890). DCA and CIC evaluations further confirmed the model’s good calibration and significant net clinical benefit.Additionally,1000 bootstrap resamplings in the entire dataset demonstrated robust diagnostic performance(AUC 0.826, 95%CI: 0.798-0.852).The nomogram maintained robust generalizability and clinical practical value across different centers, populations, and examination equipment.

**Conclusion:**

The nomogram model we developed has good diagnostic performance and provides added value for the individualized diagnosis and treatment of PTC.

## 1 Introduction

The thyroid gland is a central endocrine organ, and the global detection rate of thyroid nodules (TNs) continues to rise (1). Clinical data indicate that over 80% of TNs are benign or non-functional. Notably, up to 68.8% of surgical resection specimens are still confirmed as benign lesions (2). Overtreatment of some nodules not only damages thyroid tissue but may also lead to lifelong thyroid hormone replacement therapy, increasing the psychological burden on patients (3). PTC, accounting for 85%-90% of thyroid cancer cases, has shown a systematic increase in numerous countries over the past three decades (4). Although PTC generally has a favorable prognosis, advanced stages exhibit typical malignant biological behaviors, potentially causing severe symptoms such as dyspnea and dysphagia (5). Current differentiation between benign and malignant TNs primarily relies on ultrasound and fine-needle aspiration biopsy (FNAB) (6). However, US diagnosis is limited by operator subjectivity and equipment variability, while FNAB is invasive and may yield insufficient samples due to deep-seated or small nodules (7). With the increasing detection rate of TNs, identifying convenient, objective, and reproducible biomarkers is crucial for aiding differential diagnosis and guiding individualized treatment.

Systemic inflammatory response plays a key role in the development and progression of various malignancies (8). The lymphocyte-related composite indicators derived from inflammatory mediators, namely the Neutrophil-to-Lymphocyte Ratio (NLR), Platelet-to-Lymphocyte Ratio (PLR), and Lymphocyte-to-Monocyte Ratio (LMR), have demonstrated significant value in the diagnosis and prognostic assessment of lung cancer, liver cancer, colorectal cancer, and prostate cancer due to their ease of detection and low cost (9–15). In thyroid cancer research, these non-specific markers of thyroid inflammation have gained attention (16). However, existing studies present conflicting results, are often based on single-center data with small sample sizes, and primarily focus on their relationship with PTC prognosis, lymph node metastasis (LNM), and disease-free survival (DFS) (16–18). In 2024, Weng and colleagues (19) developed predictive models using multiple machine learning algorithms based on 6,102 participants. Random Forest and LightGBM demonstrated superior performance, with age, sex, and urinary iodine levels identified as key predictors. This study emphasized population-level early screening rather than malignancy differentiation, with the Random Forest model achieving an internal validation AUC of 0.891, highlighting the feasibility of large-scale screening using low-cost clinical data.

Review of existing literature reveals that current models predominantly rely on ultrasound characteristics or single serological biomarkers, exhibiting limited applicability in resource-limited settings. To bridge this gap, the present study integrates clinical features, ultrasound findings, and the inflammatory marker NLR into a nomogram model, aiming to achieve more precise prediction of malignancy risk in thyroid nodules.

## 2 Methods

### 2.1 Patient selection and study design

This retrospective study was approved by the Institutional Review Board of Institution A (Approval No. 2021-321), and the requirement for informed consent was waived. The inclusion criteria were as follows: 1) to ensure the accuracy and consistency of outcome variables, the final diagnosis for both the PTC group and the BTN group was confirmed by postoperative histopathology and/or immunohistochemistry, which served as the gold standard; 2) completion of thyroid ultrasound within one month prior to surgery; 3) availability of complete blood count and biochemical test results within two weeks before surgery. The exclusion criteria were as follows: 1) any prior antitumor therapy; 2) presence of severe trauma or acute infection within two weeks before surgery; 3) comorbid other malignancies or chronic consumptive diseases affecting blood cell levels; 4) diagnosis of autoimmune thyroiditis; 5) concurrent diagnosis of PTC and BTN, or BTN coexisting with papillary thyroid microcarcinoma (PTMC).

**Figure 1:**
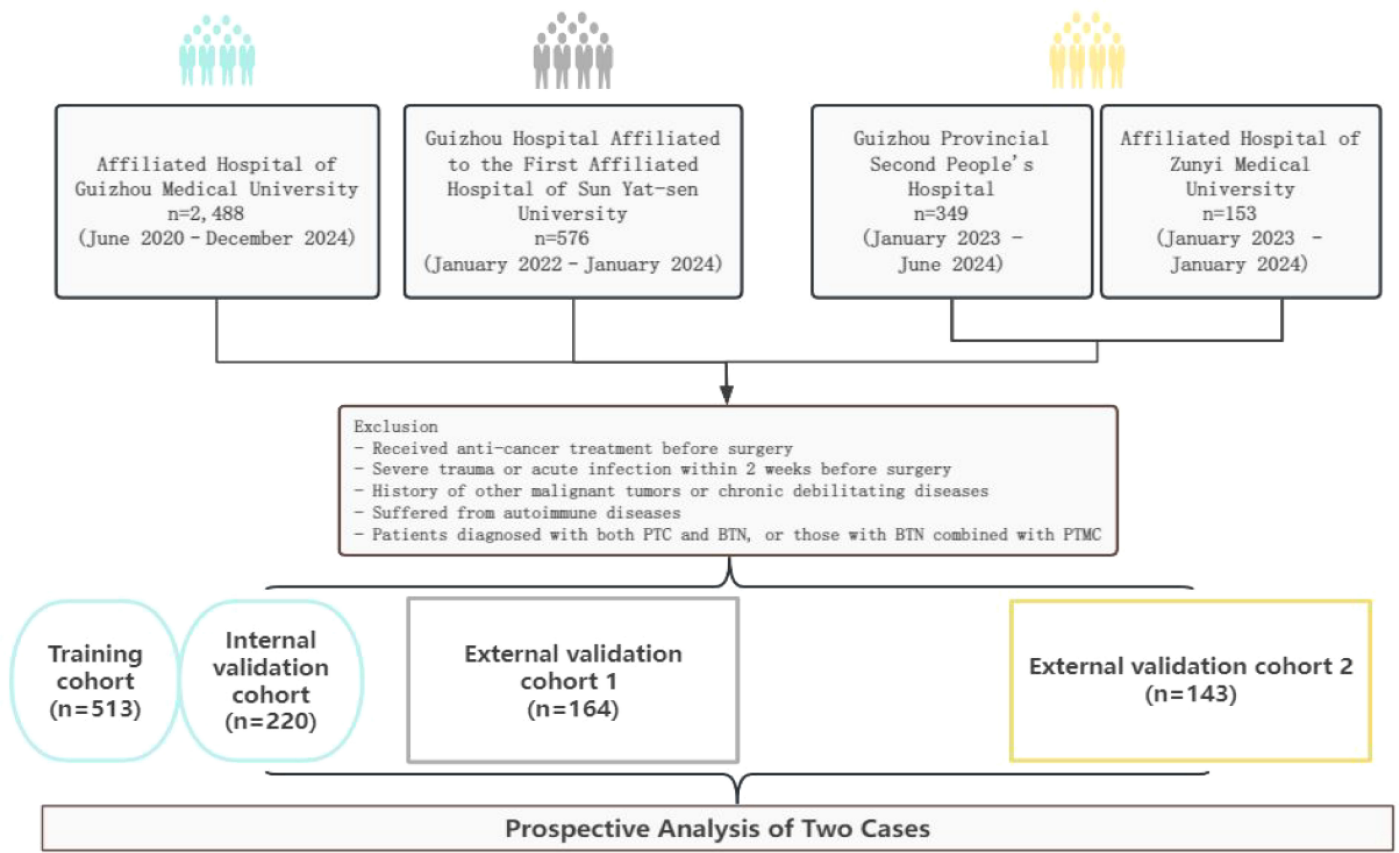
Study design and flowchart.

A total of 1040 patients (207 males and 833 females) from four medical centers were ultimately included in the final analysis. The study design and workflow are detailed in **Figure 1**.

### 2.2 Baseline data and cut-off values for inflammatory biomarkers

Baseline data included:1) Clinical characteristics: Gender(Female/Male), Age≥45years(Yes/No), Smoking(Yes/No); 2) Ultrasound imaging parameters: Tumor Dimension≥10mm(Yes/No), Morphology Irregular(Yes/No), Margin ill-defined(Yes/No), Aspect ratio≥1(A/T≥1,Yes/No), Microcalcification(Yes/No); 3) Laboratory tests: Neutrophil count (Neu), Lymphocyte count (Lym), Monocyte count (MO), and Platelet count (PLT). Subsequently, three lymphocyte-related inflammatory indices were calculated:1) NLR=Neu/Lym; 2)PLR = PLT/Lym; 3) LMR= Lym/MO. The optimal cut-off values for NLR, PLR, and LMR were determined exclusively in the training cohort using receiver operating characteristic (ROC) curve analysis. The point on the ROC curve corresponding to the maximum Youden index (Sensitivity+Specificity-1) was selected as the optimal threshold (20).

### 2.3 Conventional ultrasound examination instruments

Thyroid lesion examinations were performed by experienced sonographers using the following ultrasound systems: 1) Philips EPIQ5 (Netherlands) with an L12–5 probe (5–12 MHz); 2) Mindray Resona R9T (Shenzhen, China) with an L14–3 probe (3–14 MHz); 3) Aplio i800 (Tokyo, Japan) with an L14–3 probe (3–14 MHz); 4) GE Voluson E8 (GE Healthcare, USA) with an ML6–15 probe (6–15 MHz); 5) Esaote (Italy) with an L4–15 probe (4–15 MHz).

### 2.4 Imaging evaluation and consistency verification

All images were independently and blindly assessed by two sonographers: first by an attending sonographer (Y.Y.) with 10 years of experience, and then by a senior sonographer (Y.C.) with 16 years of experience. Inter-observer consistency was evaluated using a two-way random-effects model to calculate intraclass correlation coefficient(ICC). Only findings with an ICC greater than 0.75 were included in the final analysis.

We selected the following ultrasound malignant indicators for evaluation: 1)Morphology Irregular: Nodule margins presenting as spiculated, angular, or microlobulated;2)Margin ill-defined: Poorly defined boundary between the lesion and the surrounding thyroid parenchyma; 3)Microcalcification: Punctuate hyperechoic foci measuring <1 mm in diameter, with or without posterior acoustic shadowing; 4) A/T≥1: The anteroposterior diameter of the nodule is greater than its transverse or longitudinal diameter on either transverse or longitudinal sections, indicating a vertical growth orientation. The quantitative indicator was the Tumor Dimension, measured as the longest axis of the nodule, with a second perpendicular diameter recorded for reference (21). The characteristic ultrasound images and the ICC evaluation are shown in **Supplementary Figure 1** and **Supplementary Table 1**.

### 2.5 Feature selection and model evaluation

Univariate logistic regression was performed on all baseline characteristics, and variables with *P* < 0.05 were included in multivariate logistic regression. Variables retaining statistical significance (*P* < 0.05) in the multivariate analysis were used to construct the final nomogram.

Discrimination was assessed by the area under the ROC curve (AUC), with the optimal cutoff determined by the maximum Youden index. Sensitivity, specificity, positive predictive value (PPV), negative predictive value (NPV), accuracy, and F1 score were calculated accordingly. Calibration was evaluated using the Hosmer-Lemeshow test, Brier score, and calibration curves. Robustness was assessed via bootstrap validation (1,000 resamples) to obtain bias-corrected AUC and 95% confidence intervals (CIs).Clinical utility was assessed using DCA and CIC.

### 2.6 Statistical analysis

All statistical analyses were performed using R software version 4.1.2 and SPSS version 22.0. Continuous variables are presented as mean ± standard deviation. For comparisons between groups, the t-test was used for data that were normally distributed and had homogeneous variances, while the Mann-Whitney U test was used for data that did not meet these criteria. Categorical variables were compared between groups using the χ2 test or Fisher’s exact test. Intra-class correlation coefficients (ICCs) were used to assess inter-observer consistency: moderate (0.5 ≤ICC<0.75), good (0.75≤ ICC<0.9), and excellent (ICC≥0.9) (22). The following R packages were used: rms (for nomogram and calibration curve construction) and rmda (for decision curve and clinical impact curve analysis). A two-sided p-value of less than 0.05 was considered statistically significant.

## 3 Results

### 3.1 Baseline characteristics and cut-off values of inflammatory biomarkers

A total of 1040 patients with PTC and BTN were included in the final analysis. The patient sources and cohort allocation were as follows: 733 patients from Institution A were randomly divided into a training cohort (n=513; recruited from June 2020 to June 2024) and an internal validation cohort (n=220; recruited from December 2023 to December 2024) in a 7:3 ratio. Patients from Institution B constituted external validation cohort 1 (n=164; recruited from January 2022 to January 2024), while patients from Institutions C and D were combined as external validation cohort 2 (n=143; recruited from January 2023 to June 2024). The baseline characteristics of the patients in each cohort are presented in **Table 1**. The definitions and determined cut-off values for the inflammatory biomarkers are detailed in **Supplementary Table 2**.

**Table 1:** Baseline characteristics of patients in different cohorts.

Variables	Training Set			Internal validation cohort			External validation cohort 1			External validation cohort 2		
	BTN	PTC	*p values*	BTN	PTC	*p values*	BTN	PTC	*p values*	BTN	PTC	*p values*
	n=188	n=325		n=71	n=149		n=55	n=109		n=49	n=94	
Clinical+Ultrasound Markers												
Sex			0.458			0.265			0.051			0.049
Female	154 (81.9%)	256 (78.8%)		58 (81.7%)	110 (73.8%)		50 (90.9%)	84 (77.1%)		46 (93.9%)	75 (79.8%)	
Male	34 (18.1%)	69 (21.2%)		13 (18.3%)	39 (26.2%)		5 (9.1%)	25 (22.9%)		3 (6.1%)	19 (20.2%)	
Age≥45years			<0.001			0.144			0.047			0.09
NO	66 (35.1%)	191 (58.8%)		28 (39.4%)	76 (51.0%)		21 (38.2%)	61 (56.0%)		18 (36.7%)	50 (53.2%)	
YES	122 (64.9%)	134 (41.2%)		43 (60.6%)	73 (49.0%)		34 (61.8%)	48 (44.0%)		31 (63.3%)	44 (46.8%)	
Smoking			0.445			0.994			0.629			1
NO	171 (91.0%)	303 (93.2%)		65 (91.5%)	138 (92.6%)		51 (92.7%)	97 (89.0%)		49 (100%)	93 (98.9%)	
YES	17 (9.0%)	22 (6.8%)		6 (8.5%)	11 (7.4%)		4 (7.3%)	12 (11.0%)		0 (0.00%)	1 (1.1%)	
A/T≥1			<0.001			<0.001			<0.001			<0.001
NO	160 (85.1%)	146 (44.9%)		63 (88.7%)	77 (51.7%)		51 (92.7%)	52 (47.7%)		48 (98.0%)	66 (70.2%)	
YES	28 (14.9%)	179 (55.1%)		8 (11.3%)	72 (48.3%)		4 (7.3%)	57 (52.3%)		1 (2.0%)	28 (29.8%)	
Morphology Irregular			<0.001			<0.001			<0.001			<0.001
NO	133 (70.7%)	89 (27.4%)		54 (76.1%)	61 (40.9%)		47 (85.5%)	29 (26.6%)		49 (100%)	31 (33.0%)	
YES	55 (29.3%)	236 (72.6%)		17 (23.9%)	88 (59.1%)		8 (14.5%)	80 (73.4%)		0 (0.00%)	63 (67.0%)	
Margin ill-defined			<0.001			<0.001			<0.001			<0.001
NO	125 (66.5%)	110 (33.8%)		53 (74.6%)	53 (35.6%)		45 (81.8%)	44 (40.4%)		47 (95.9%)	53 (56.4%)	
YES	63 (33.5%)	215 (66.2%)		18 (25.4%)	96 (64.4%)		10 (18.2%)	65 (59.6%)		2 (4.1%)	41 (43.6%)	
Microcalcification			<0.001			<0.001			<0.001			0.008
NO	125 (66.5%)	120 (36.9%)		52 (73.2%)	55 (36.9%)		44 (80.0%)	38 (34.9%)		32 (65.3%)	38 (40.4%)	
YES	63 (33.5%)	205 (63.1%)		19 (26.8%)	94 (63.1%)		11 (20.0%)	71 (65.1%)		17 (34.7%)	56 (59.6%)	
Tumor Dimension≥10mm			<0.001			<0.001			<0.001			<0.001
NO	128 (68.1%)	111 (34.2%)		52 (73.2%)	52 (34.9%)		39 (70.9%)	35 (32.1%)		47 (95.9%)	44 (46.8%)	
YES	60 (31.9%)	214 (65.8%)		19 (26.8%)	97 (65.1%)		16 (29.1%)	74 (67.9%)		2 (4.1%)	50 (53.2%)	
Inflammatory Markers												
Lymphocyte count(10^9/L)	1.75 (0.46)	1.81 (0.47)	0.133	1.83 (0.49)	1.84 (0.49)	0.851	1.75 (0.40)	1.82 (0.55)	0.39	1.82 (0.62)	1.86 (0.78)	0.729
Monocyte count(10^9/L)	0.39 (0.12)	0.41 (0.16)	0.242	0.40 (0.12)	0.42 (0.21)	0.38	0.31 (0.13)	0.34 (0.11)	0.115	0.36 (0.13)	0.42 (0.27)	0.08
Neutrophil count(10^9/L)	3.56 (1.38)	3.79 (1.20)	0.065	3.45 (1.11)	3.56 (1.13)	0.467	3.42 (1.57)	4.10 (1.46)	0.009	3.34 (1.27)	3.40 (1.46)	0.811
Platelets(10^9/L)	233 (66.0)	238 (60.9)	0.402	234 (63.0)	231 (64.1)	0.803	234 (55.5)	235 (62.6)	0.9	234 (62.8)	237 (60.7)	0.824
NLR≥1.83			0.025			0.46			<0.001			0.17
NO	88 (46.8%)	118 (36.3%)		35 (49.3%)	64 (43.0%)		29 (52.7%)	16 (14.7%)		27 (55.1%)	39 (41.5%)	
YES	100 (53.2%)	207 (63.7%)		36 (50.7%)	85 (57.0%)		26 (47.3%)	93 (85.3%)		22 (44.9%)	55 (58.5%)	
LMR≥4.08			0.237			1			0.073			1
NO	70 (37.2%)	103 (31.7%)		20 (28.2%)	42 (28.2%)		8 (14.5%)	6 (5.5%)		13 (26.5%)	25 (26.6%)	
YES	118 (62.8%)	222 (68.3%)		51 (71.8%)	107 (71.8%)		47 (85.5%)	103 (94.5%)		36 (73.5%)	69 (73.4%)	
PLR ≤ 93.13			0.101			0.292			0.539			1
NO	168 (89.4%)	272 (83.7%)		62 (87.3%)	120 (80.5%)		50 (90.9%)	102 (93.6%)		40 (81.6%)	77 (81.9%)	
YES	20 (10.6%)	53 (16.3%)		9 (12.7%)	29 (19.5%)		5 (9.1%)	7 (6.4%)		9 (18.4%)	17 (18.1%)	

PTC, Papillary Thyroid Carcinoma;BTN, Benign thyroid nodule;A/T ratio≥1, Anteroposterior-to-Transverse diameter ratio≥1; NLR, Neutrophil-to-Lymphocyte Ratio; PLR, Platelet-to-Lymphocyte Ratio;LMR, Lymphocyte-to-Monocyte Ratio.

### 3.2 Inter-observer agreement of qualitative ultrasonic parameters

The two sonologists demonstrated excellent agreement in the assessment of Tumor Dimension [ICC = 0.964 (95%CI:0.945-0.984)], Margin ill-defined [ICC = 0.959 (95%CI:0.938-0.979)], Microcalcification [ICC = 0.945 (95%CI:0.922-0.969)], A/T≥1[ICC = 0.938 (95%CI:0.912-0.963)]. Good agreement was observed for the evaluation of Morphology Irregular [ICC = 0.798 (95%CI:0.755-0.842)].

### 3.3 Feature selection

Univariate logistic regression analysis of the 16 baseline characteristics in the internal training set initially identified 7 candidate variables. These were subsequently entered into a multivariate logistic regression model, which ultimately identified 6 independent predictors for PTC in **Table 2**. The results showed that Age≥45years (OR = 0.357; 95%CI, 0.225-0.564; *P* < 0.001), NLR≥1.83 (OR = 1.656; 95%CI, 1.049-2.614; *P* = 0.03), Morphology Irregular(OR = 3.810;95%CI, 2.357-6.159; *P* < 0.001), A/T≥1 (OR = 3.942; 95%CI, 2.293-6.776; *P* < 0.001),Microcalcification (OR = 2.261; 95%CI, 1.437-3.556; *P* < 0.001), and Tumor Dimension≥10mm (OR = 1.866; 95%CI, 1.142-3.048; *P* = 0.013) were independent predictive factors for differentiating PTC from BTN. A heatmap illustrating the correlation coefficients among these six selected features is provided in **Supplementary Figure 2**.

**Table 2:** Feature selection.

Factor			Univariate analysis		Multivariate analysis	
			OR (95%CI)	*P values*	OR (95%CI)	*P values*
Clinical+Ultrasound Markers	Sex	(Female/Male)	1.221(0.773-1.928)	0.392		
	Age	(≥45years/<45years)	0.380(0.262-0.551)	<0.001	0.357(0.225-0.564)	<0.001
	Smoking	(Yes/No)	0.730(0.377-1.413)	0.351		
	A/T ratio≥1	(Yes/No)	7.006(4.435-11.066)	<0.001	3.942(2.293-6.776)	<0.001
	Morphology irregular	(Yes/No)	6.412(4.308-9.544)	<0.001	3.810(2.357-6.159)	<0.001
	Margin ill-defined	(Yes/No)	3.878(2.652-5.672)	<0.001	1.355(0.825-2.226)	0.230
	Microcalcification	(Yes/No)	3.390(2.324-4.944)	<0.001	2.261(1.437-3.556)	<0.001
	Tumor dimension	(≥10mm/<10mm)	4.113(2.805-6.032)	<0.001	1.866(1.142-3.048)	0.013
Inflammatory Markers	Lymphocyte count	(10^9/L)	1.343(0.911-1.981)	0.137		
	Monocyte count	(10^9/L)	2.134(0.540-8.428)	0.280		
	Neutrophil count	(10^9/L)	1.155(0.996-1.339)	0.057		
	Platelet count	(10^9/L)	1.001(0.998-1.004)	0.391		
	NLR	(≥1.83/<1.83)	1.544(1.072-2.224)	0.020	1.656(1.049-2.614)	0.030
	LMR	(≥4.08/<4.08)	1.279(0.877-1.864)	0.201		
	PLR	(≤93.13/>93.13)	1.637(0.945-2.834)	0.079		

OR, Odds Ratio;CI, Confidence Interval;A/T ratio≥1, Anteroposterior-to-Transverse diameter ratio≥1; NLR, Neutrophil-to-Lymphocyte Ratio ;PLR, Platelet-to-Lymphocyte Ratio;LMR, Lymphocyte-to-Monocyte Ratio.

### 3.4 Model construction

**Figure 2:**
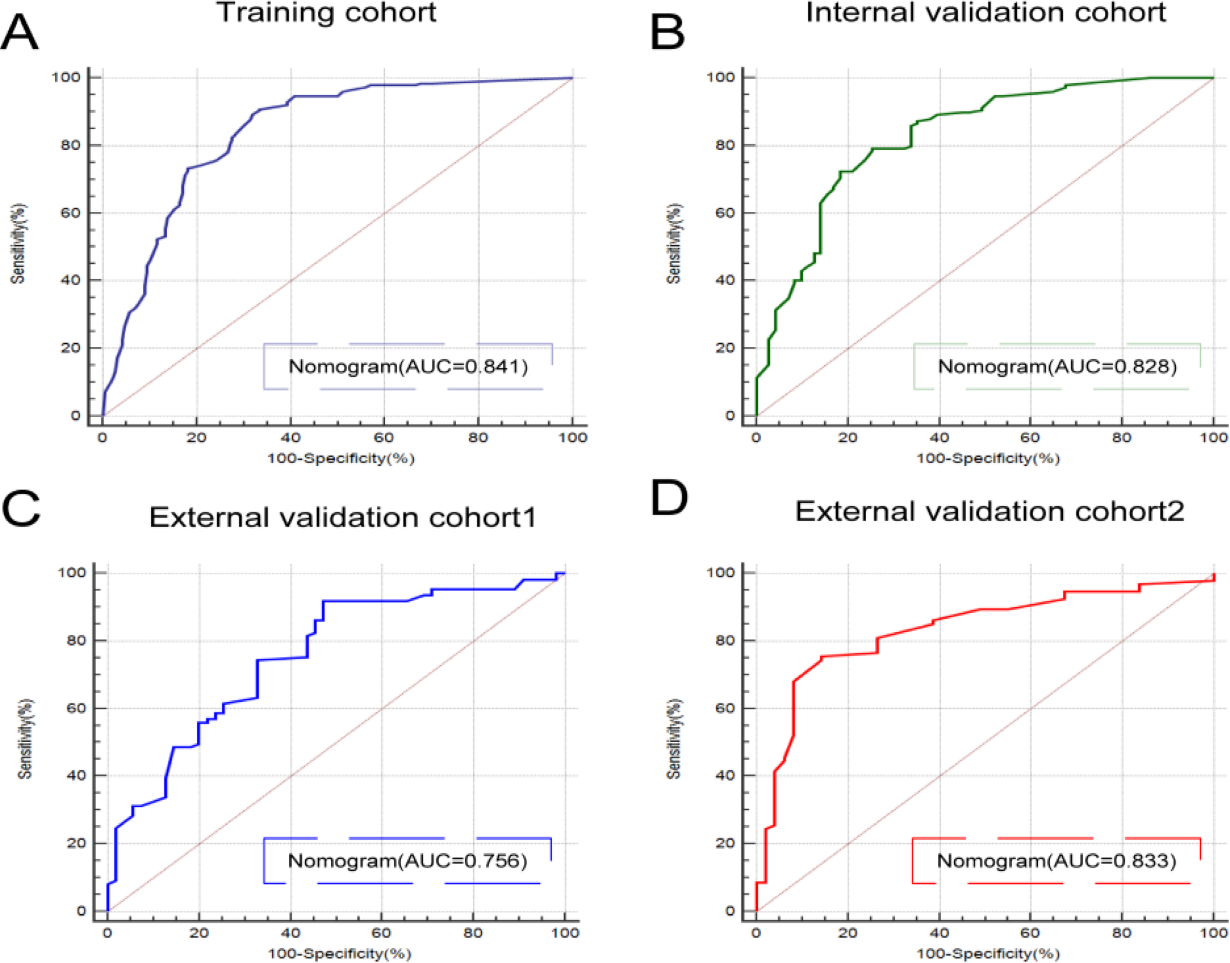
**(A-D)** ROC curves and AUC values of the nomogram model in the training cohort **(A)**, internal validation cohort **(B)**, external validation cohort 1 **(C)**, and external validation cohort 2 **(D)**.

**Figure 3:**
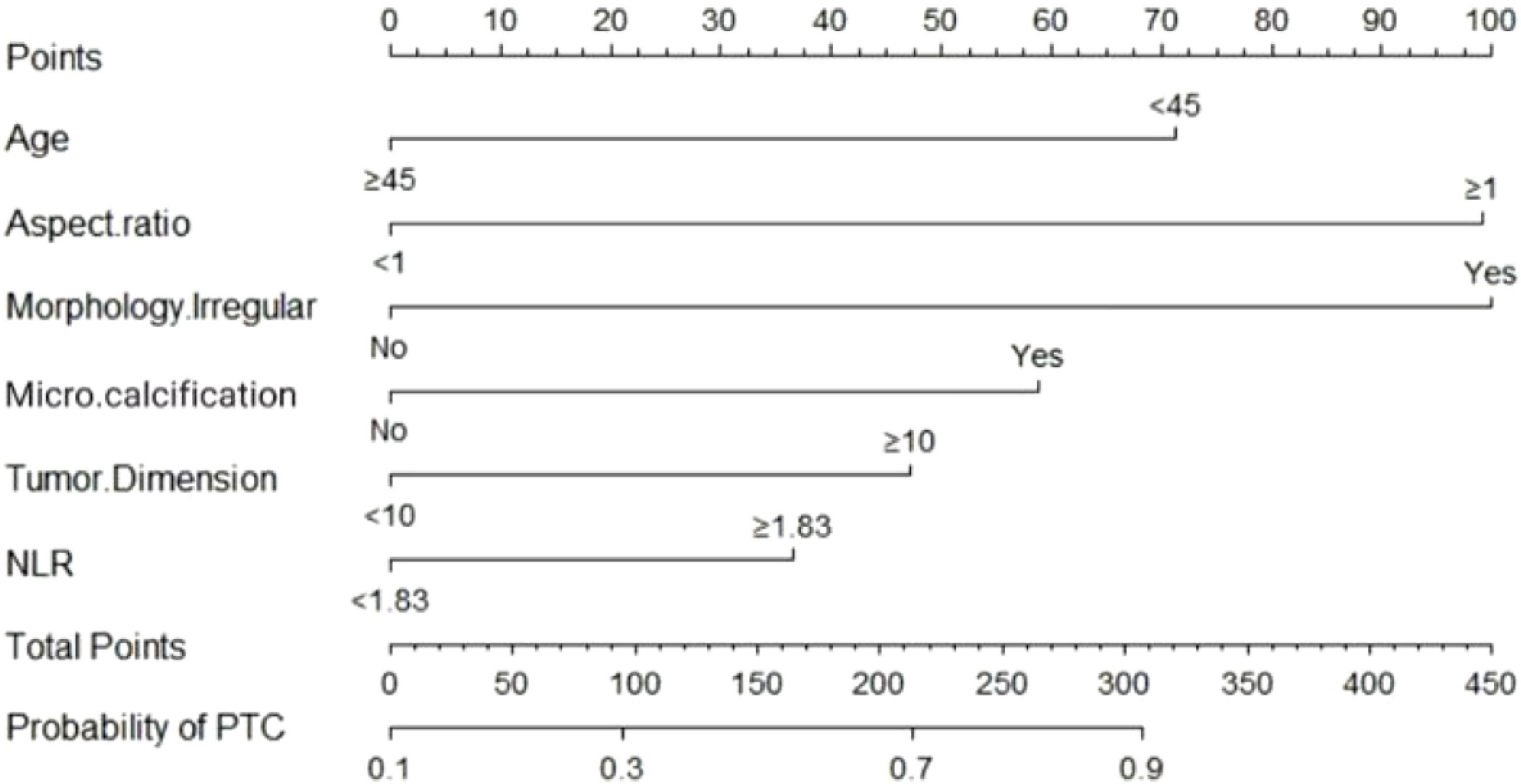
Nomogram.

The nomogram showed excellent predictive performance in the training, internal validation, and external validation cohort 2 (AUCs: 0.841, 0.828, and 0.833, respectively), but performed moderately in external validation cohort 1 (AUC: 0.756) (**Figure 2**). It significantly outperformed all single predictors in discriminating PTC from BTN (Delong test, **Supplementary Table 3**). **Figure 3** illustrates the nomogram structure, and the predictive formula is: Nomogram=-1.181-1.021×Age+1.421×Aspect ratio+1.433×Irregular morphology+0.842×Microcalcification+0.675×Tumor dimension+0.523×NLR.

### 3.5 Model evaluation and validation

**Figure 4:**
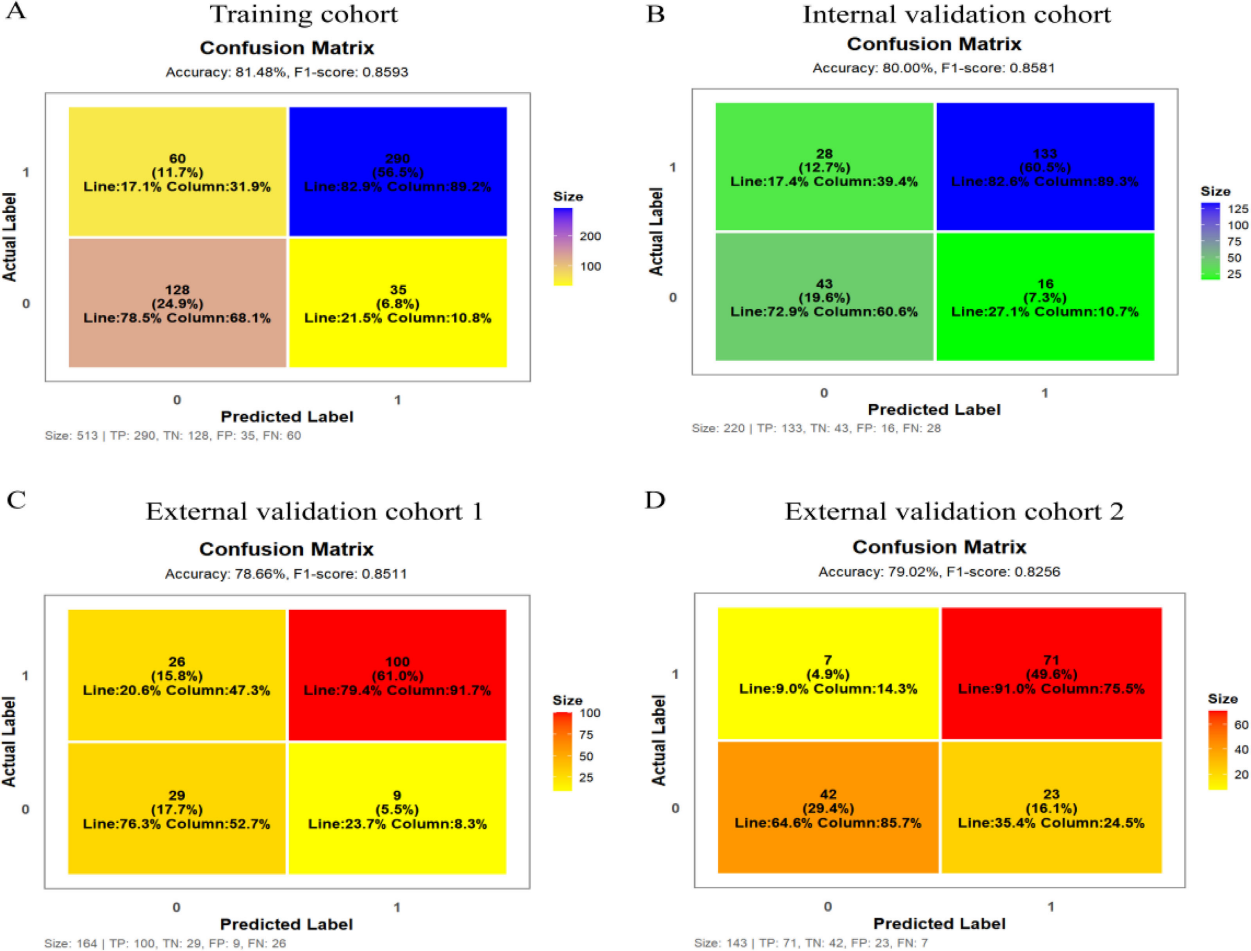
Confusion matrix.

**Figure 5:**
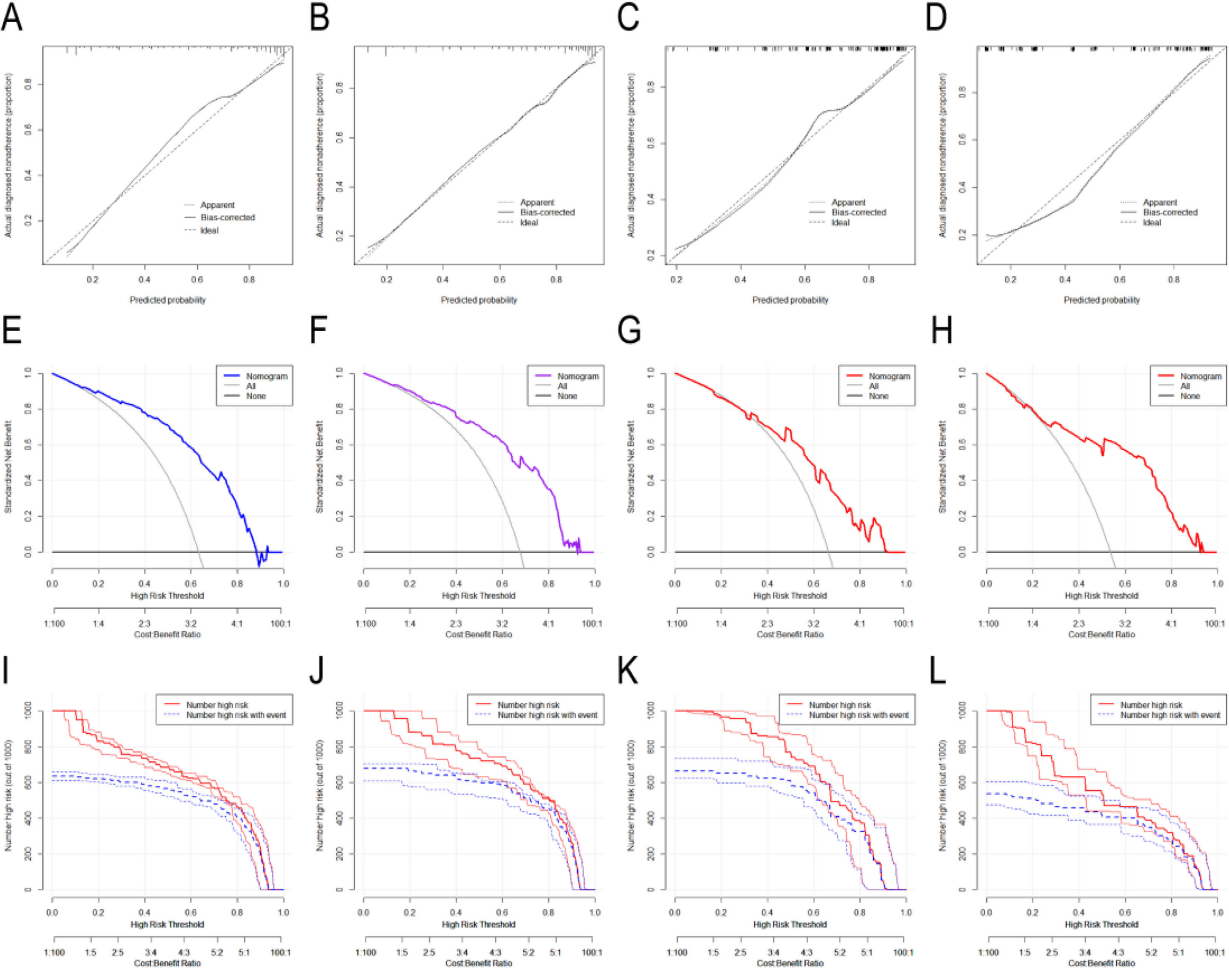
**(A-L)** The figure presents the calibration curves, Decision Curve Analysis (DCA), and Clinical Impact Curves (CIC) for predicting papillary thyroid carcinoma (PTC) across the training cohort **(A, E, I)**, internal validation cohort **(B, F, J)**, external validation cohort 1 **(C, G, K)**, and external validation cohort 2 **(D, H, L)**. Visual analysis of the DCA demonstrates that the model achieves favorable clinical net benefit within specific probability threshold ranges across all four cohorts (training cohort: 15%–85%; internal validation cohort: 18%-95%; external validation cohort 1: 35%–95%; external validation cohort 2: 25%–95%). Furthermore, the CIC analysis substantiates the model’s excellent clinical feasibility and superior performance characteristics.

The nomogram demonstrated favorable discrimination across the training, internal and external validation cohorts (**Table 3**, **Figure 4**), while the detailed performance of the six individual features in each cohort is presented in **Supplementary Table 4**. Calibration curves showed excellent agreement with histopathological outcomes (**Figures 5A–D**); Decision curve analysis indicated superior net clinical benefit across clinically relevant thresholds (**Figures 5E–H**); While clinical impact curves further validated its robustness and clinical utility (**Figure 5I–L**).

**Table 3:** The specific diagnostic performance of the nomogram model in the four cohorts.

Different cohorts (Nomogram)	Training cohort (n = 513)	Internal validation cohort (n = 220)	External validation cohort 1 (n = 164)	External validation cohort2 (n = 143)
Accuracy	0.815	0.800	0.787	0.790
AUC	0.841	0.828	0.756	0.833
95% CI	0.807-0.872	0.772-0.876	0.683-0.820	0.762-0.890
Sensitivity	0.829	0.826	0.794	0.910
Specificity	0.785	0.729	0.763	0.646
PPV	0.892	0.893	0.917	0.755
NPV	0.681	0.606	0.527	0.857
F1-Score	0.859	0.858	0.851	0.826
Brier-Score	0.146	0.148	0.181	0.161

AUC, Confidence Interval;CI, Confidence Interval;PPV, Positive Predictive Value;NPV, Negative Predictive Value.

### 3.6 Bootstrap validation (1000 iterations)

**Figure 6:**
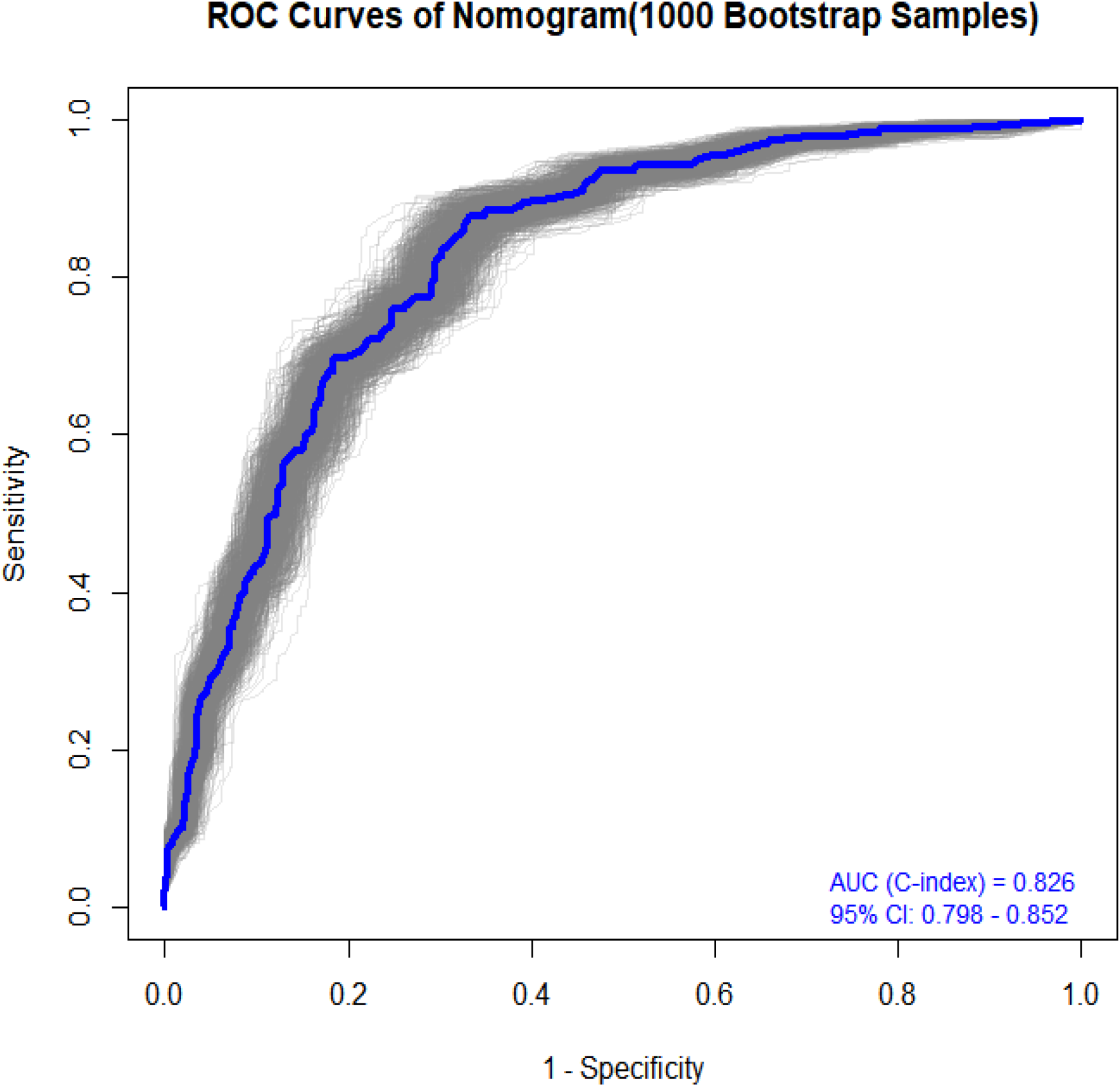
Bootstrap validation (1000 iterations).

To assess potential instability arising from random data splitting, we performed bootstrap validation with 1000 resamplings across the entire dataset to evaluate the nomogram’s predictive performance for preoperative PTC. Notably, the analysis demonstrated a robust AUC of 0.826 (95% CI: 0.798-0.852), as illustrated in **Figure 6**.

### 3.7 Prospective validation

**Figure 7:**
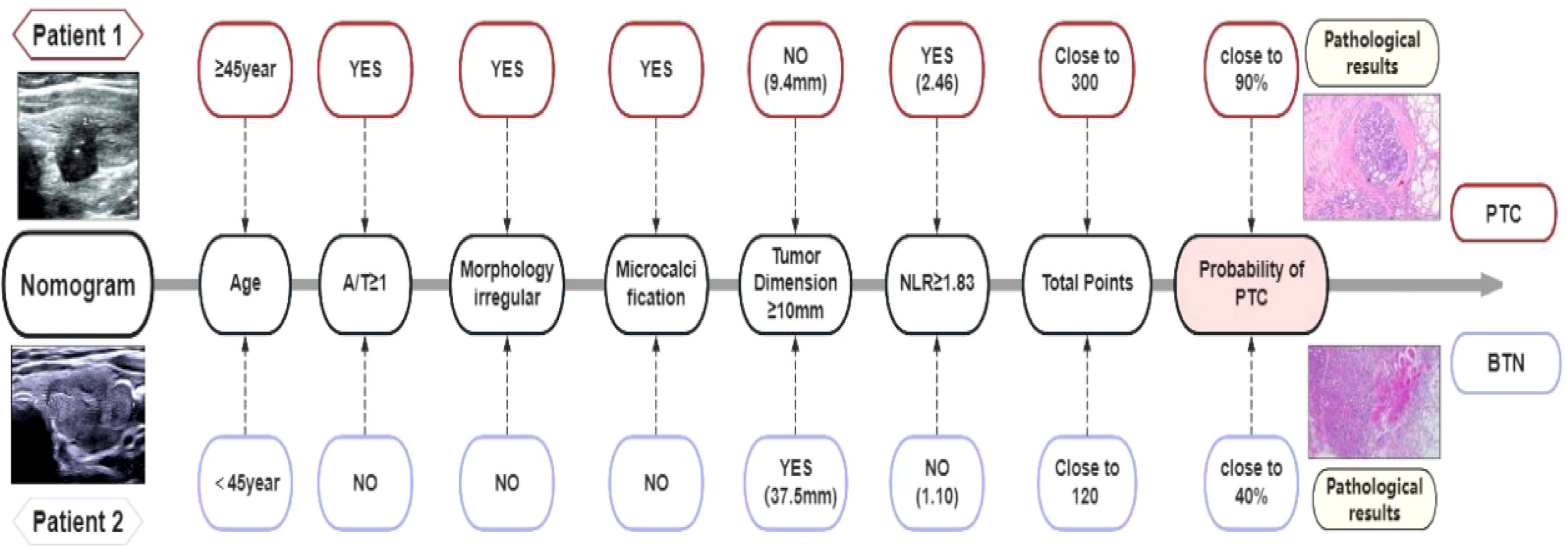
Two patients underwent prospective validation: (1) A 59-year-old male with an 8-mm irregular hypoechoic nodule in the left thyroid lobe (A/T≥1, microcalcifications), preoperative NLR 2.46 (≥1.83). Nomogram score: ~300 points (~90% PTC probability). Postoperative pathology confirmed PTC. (2) A 30-year-old female with a 37.5-mm regular cystic-solid nodule in the left thyroid lobe (A/T < 1, no microcalcifications), preoperative NLR 1.10 (<1.83). Nomogram score: ~120 points (~40% PTC probability). Pathology confirmed BTN.

We prospectively validated the nomogram using two independent patient cases, both of which showed a high concordance between the nomogram output and the final pathological diagnosis. Consequently, the nomogram was established as the optimal predictive tool for assessing the risk of PTC in **Figure 7**.

## 4 Discussion

We developed a nomogram model combining NLR with clinical-ultrasound features, which demonstrated stable diagnostic performance across multiple independent cohorts.

Our nomogram model shows that: Regarding patient age, Age≥45 years was significantly associated with a reduced risk of PTC. This may be attributed to a higher incidence of fusion genes in younger patients, potentially due to more active thyroid cell division and relatively insufficient DNA repair capacity before puberty. This makes chromosomes more susceptible to translocations induced by factors such as ionizing radiation, thereby promoting the formation of oncogenic fusion genes (23, 24), which aligns with previous findings (25). Among the US characteristics: Morphology Irregularr, Microcalcification and A/T≥1 were identified as independent risk factors for PTC, each contributing differently to the diagnostic performance of the nomogram. Malignant tumor cells, characterized by unlimited proliferation and invasive growth, often lead to masses with irregular shapes and ill-defined margins (26). The vigorous metabolism of cancer cells results in the release of acidic metabolites during proliferation, which can cause local deposition of calcium salts, forming microcalcifications with high diagnostic specificity (6). A/T≥1 suggests the infiltrative nature of malignant cells, manifesting as vertical growth of the nodule on planes perpendicular to the ultrasound beam (27). Notably, our study found that Tumor Dimension≥10mm was also an independent risk factor for predicting PTC. As suggested by Meng C et al (28), TNs≥10mm have a significantly higher probability of lymph node metastasis and early tumor infiltration events compared to those <10mm, indicating that tumor volume may, to some extent, hint at its invasive potential.

Regardless of whether systemic inflammation plays a pro-tumor or anti-tumor role in the tumor microenvironment, accumulating evidence indicates that derivative indicators of peripheral blood inflammatory cells are closely associated with the malignant potential and poor prognosis of various solid tumors, including thyroid cancer (29–31). Inflammatory cells can indirectly promote carcinogenesis by disrupting cellular structures and DNA, while tumor cells can also secrete pro-inflammatory factors to recruit inflammatory cells into the microenvironment (32). Among these indicators, an elevated NLR reflects an absolute increase in peripheral neutrophils and/or an absolute decrease in lymphocytes. Neutrophils can drive tumor cells into the cell cycle and enhance their metastatic capacity (33). In our nomogram model, NLR can serve as an auxiliary parameter for assessing the benign or malignant nature of thyroid nodules. Using a cut-off value of 1.83, NLR≥1.83 was an independent risk factor for predicting PTC. An elevated NLR was associated with the occurrence of PTC, which is very close to the cut-off value of 1.73 reported by Yildiz EO et al (34). They reported that at this threshold, NLR had a sensitivity of 51.77% for discriminating malignant nodules, a specificity of 86.15% for distinguishing benign nodules, and an overall diagnostic accuracy of 73.3% for malignant nodules. In fact, Seretis C et al (35) first pointed out in 2013 that the preoperative NLR was higher in patients with PTMC or Thyroid Carcinoma (TC) compared to patients with benign nodules and controls. Our findings are consistent with this, indicating an increased risk of PTC with elevated NLR. Our analysis across four multicenter cohorts showed that when NLR was used alone to predict PTC, the AUC values were 0.553, 0.532, 0.690, and 0.568, respectively, which were significantly lower than the diagnostic performance of the nomogram model (AUCs of 0.841, 0.828, 0.756, and 0.833, respectively). Incorporating NLR into the nomogram model further improved the predictive accuracy for PTC risk. Therefore, this study suggests that NLR is more suitable as an auxiliary parameter within a comprehensive predictive model rather than an independent diagnostic criterion. For thyroid nodules with indeterminate ultrasound findings, integrating NLR into the nomogram for comprehensive assessment might be more beneficial for predicting the risk of PTC when deciding whether to perform FNAB.

Compared to NLR, neither LMR nor PLR demonstrated preoperative diagnostic value for PTC in this study. When a cut-off value of 93.13 was set for PLR, the OR for PLR ≤ 93.13 was 1.637 (95% CI 0.945–2.834, P > 0.05), indicating it could not effectively distinguish PTC from benign nodules. In fact, conclusions from previous studies have been inconsistent: Yildiz S et al (36) reported that PLR was significantly elevated in PTC patients and could be used to differentiate PTC from nodular goiter; whereas Ozmen S et al (31) suggested that PLR is more suitable for assessing the risk of metastasis or recurrence rather than early diagnosis. Other scholars have pointed out that the biological function of platelets depends more on the Mean Platelet Volume (MPV) than on the absolute platelet count (37), which might help explain the heterogeneity in PLR results across different studies. Similarly, when the LMR cut-off was set at 4.08, the OR for LMR ≥ 4.08 was only 1.279 (95% CI 0.877-1.864, P = 0.201), also lacking significant preoperative predictive value. Although LMR showed limited performance in early diagnosis, several studies suggest it might be superior to NLR for prognostic assessment (38, 39). Team Cai Y (16) indicated that a low preoperative LMR was closely associated with an increased risk of PTC recurrence, decreased overall survival, and more aggressive pathological features; conversely, a higher LMR predicted a favorable prognosis.

For cytologically indeterminate thyroid nodules, the nomogram may serve as an adjunctive risk-stratification tool, assisting clinicians in balancing active surveillance against diagnostic fine-needle aspiration. Additionally, this model provides quantitative supplementation to traditional ultrasound features, potentially reducing unnecessary surgery arising from subjective interpretive variability, particularly when managing borderline or suspicious lesions.

Although our nomogram demonstrated promising diagnostic performance, several limitations warrant consideration. First, this retrospective study did not directly compare our model against established ultrasound risk stratification systems (ACR TI-RADS/EU-TIRADS). Our nomogram was designed not to replace these standards, but to provide complementary multimodal support by integrating clinical features with the cost-effective inflammatory marker NLR. Second, the lack of standardized ultrasound acquisition protocols across devices may introduce measurement bias. Additionally, excluding patients with concurrent thyroiditis—while reducing confounding—limits the model’s applicability to this common subgroup. Third, as a transient inflammatory marker, NLR is susceptible to fluctuations from acute infections, medications, or stress states, potentially affecting stability and reproducibility across settings.

Future research will focus on three directions. First, validating the model in thyroiditis-inclusive cohorts. Second, conducting large-scale, multicenter prospective studies to confirm efficacy and generalizability. Third, investigating the prognostic value of NLR and other inflammatory markers in PTC patients.

## 5 Conclusion

In summary, the nomogram integrating NLR with clinical-Ultrasound features provides a reliable tool for non-invasive, cost-effective, and convenient personalized preoperative risk assessment of PTC versus BTN. It holds promise as an important reference for precise diagnosis and treatment decision-making.

## Data Availability

The original contributions presented in the study are included in the article/**Supplementary Material**. Further inquiries can be directed to the corresponding author.
