# Supplementary material for: Nomogram model for predicting papillary thyroid carcinoma based on clinical-ultrasound characteristics and inflammatory biomarkers: a multicenter study
Source: Front Oncol. 2026 Apr 13;16:1752376. doi: 10.3389/fonc.2026.1752376 (PMC13110939; doi:10.3389/fonc.2026.1752376)
Supplement: Supplementary file 1 [file Table1.docx]

**Supplementary Materials**


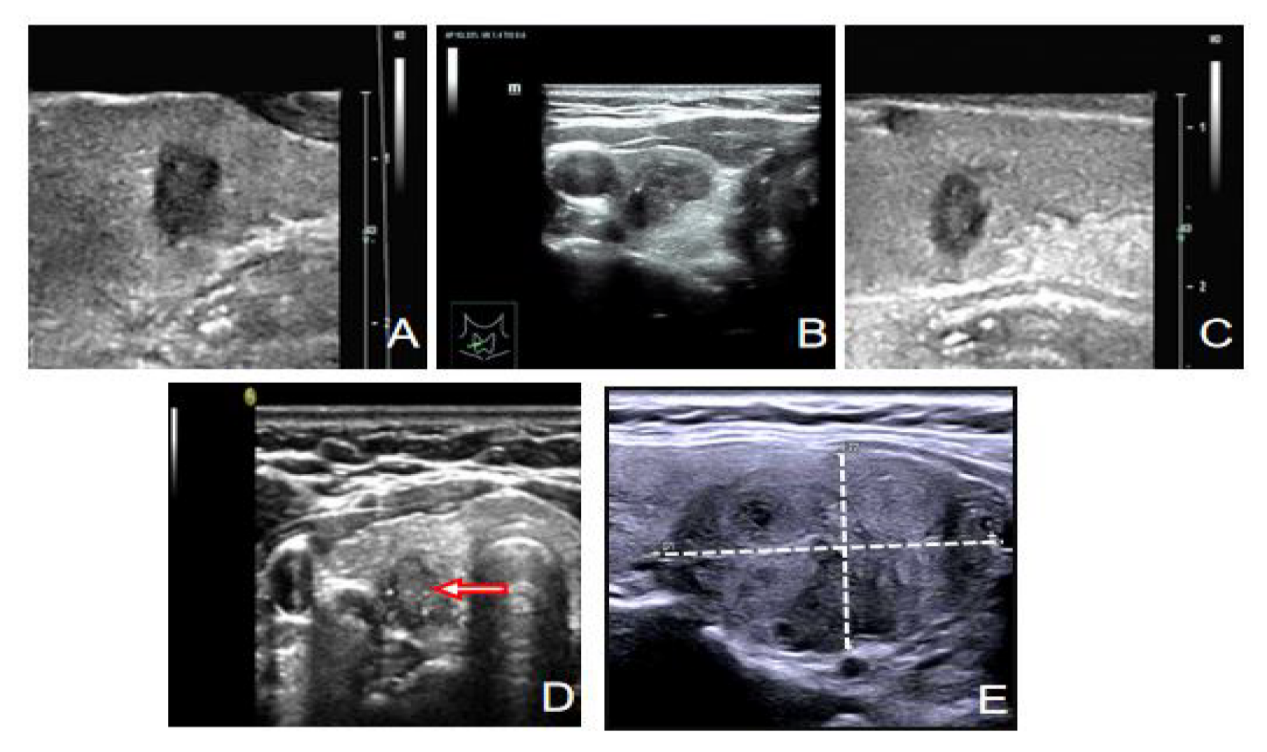


**Supplementary FIG 1:**Conventional ultrasound images of thyroid nodules: (A) Morphology Irregularr; (B) Margin ill-defined; (C) Aspect ratio≥1; (D) Microcalcification; (E) Lesion measurement.


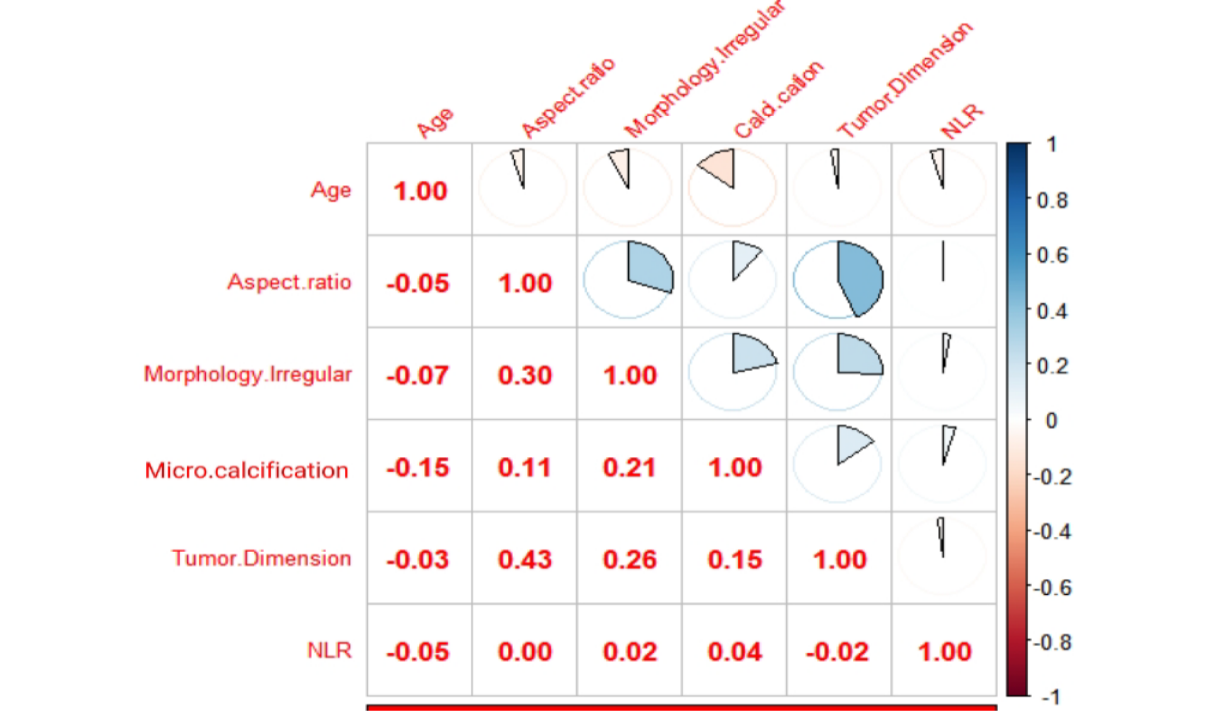


**Supplementary FIG 2:** Heatmap analysis of covariates: The heatmap analysis shows that all coefficients are below 0.45, indicating no significant multicollinearity issues in the dataset and good independence of features.

**Supplementary Table 1**

Table of inter-observer consistency analysis between two ultrasound physicians

| **Variables** | **Weighted Kappa** | **95%CI** |
| --- | --- | --- |
| Margin ill-deﬁned | 0.959 | 0.938-0.979 |
| Microcalcification | 0.945 | 0.922-0.969 |
| Morphology irregular | 0.798 | 0.755-0.842 |
| Tumor diamension | 0.964 | 0.945-0.984 |
| A/T≥1 | 0.938 | 0.912-0.963 |

A/T ratio≥1，anteroposterior-to-transverse diameter ratio；CI, Confidence Interva；Kappa，Cohen's kappa test

**Supplementary Table 2 Definitions and cutoff values of NLR, PLR, and LMR**

|  |  |  |
| --- | --- | --- |
| **Inflammatory biomarkers** | **Definition Formulas** | **Cutoff Values** |
| NLR | Neutrophil-to-lymphocyte ratio (Neu/Lym) | 1.83 |
| PLR | Platelet-to-lymphocyte ratio (PLT/Lym) | 93.13 |
| LMR | Lymphocyte-to-monocyte ratio (Lym/MO) | 4.08 |

**Supplementary Table 3**

**The nomogram model shows significantly better identification ability for PTC and BTN compared to single indicators (Delong test).**

|  |  |  |
| --- | --- | --- |
| **Different cohorts** | **Z** | ***P values*** |
| **Training cohort** | | |
| Nomogram vs.NLR≥1.83 | 10.278 | ＜0.0001 |
| Nomogram vs.Age≥45year | 9.042 | ＜0.0001 |
| Nomogram vs.Morphology irregular | 7.010 | ＜0.0001 |
| Nomogram vs.Microcalcification | 8.771 | ＜0.0001 |
| Nomogram vs.A/T≥1 | 8.415 | ＜0.0001 |
| Nomogram vs.Tumor diamension＞10mm | 8.355 | ＜0.0001 |
| **Internal Validation cohort** | | |
| Nomogram vs.NLR≥1.83 | 6.646 | ＜0.0001 |
| Nomogram vs.Age≥45year | 6.358 | ＜0.0001 |
| Nomogram vs.Morphology irregular | 6.203 | ＜0.0001 |
| Nomogram vs.Microcalcification | 4.302 | ＜0.0001 |
| Nomogram vs.A/T≥1 | 4.994 | ＜0.0001 |
| Nomogram vs.Tumor diamension＞10mm | 4.584 | ＜0.0001 |
| **External Validation cohort1** | | |
| Nomogram vs.NLR≥1.83 | 1.269 | 0.2044 |
| Nomogram vs.Age≥45year | 3.812 | 0.0001 |
| Nomogram vs.Morphology irregular | 0.650 | 0.5157 |
| Nomogram vs.Microcalcification | 0.694 | 0.4875 |
| Nomogram vs.A/T≥1 | 0.770 | 0.4415 |
| Nomogram vs.Tumor diamension＞10mm | 1.430 | 0.1528 |
| **External Validation cohort2** | | |
| Nomogram vs.NLR≥1.83 | 5.101 | ＜0.0001 |
| Nomogram vs.Age≥45year | 6.043 | ＜0.0001 |
| Nomogram vs.Morphology irregular | 4.015 | 0.0001 |
| Nomogram vs.Microcalcification | 3.920 | 0.0001 |
| Nomogram vs.A/T≥1 | 4.803 | ＜0.0001 |
| Nomogram vs.Tumor diamension＞10mm | 2.274 | 0.023 |
| NLR，Neutrophil-to-Lymphocyte Ratio；A/T ratio≥1，anteroposterior-to-transverse diameter ratio |  |  |

**Supplementary Table 4**

| **Comparison of efficacy across cohorts table** | | | |
| --- | --- | --- | --- |
| **Different cohorts** | **AUC** | **SE** | **95% CI** |
| **Training cohort** |  |  |  |
| Nomogram | 0.841 | 0.0194 | 0.807-0.872 |
| NLR≥1.83 | 0.553 | 0.0226 | 0.508-0.596 |
| Age≥45year | 0.618 | 0.0222 | 0.575-0.661 |
| Morphology irregular | 0.717 | 0.0207 | 0.676-0.755 |
| Microcalcification | 0.648 | 0.0219 | 0.605-0.689 |
| A/T ratio≥1 | 0.701 | 0.0190 | 0.659-0.740 |
| Tumor diamension≥10mm | 0.670 | 0.0215 | 0.627-0.710 |
| **Internal Validation cohort** |  |  |  |
| Nomogram | 0.828 | 0.0307 | 0.772-0.876 |
| NLR≥1.83 | 0.532 | 0.0361 | 0.463-0.599 |
| Age≥45year | 0.558 | 0.0357 | 0.490-0.625 |
| Morphology irregular | 0.676 | 0.0325 | 0.609-0.737 |
| Microcalcification | 0.682 | 0.0331 | 0.616-0.743 |
| A/T ratio≥1 | 0.685 | 0.0279 | 0.619-0.746 |
| Tumor diamension≥10mm | 0.692 | 0.0329 | 0.626-0.752 |
| **External Validation cohort 1** |  |  |  |
| Nomogram | 0.756 | 0.0401 | 0.683-0.820 |
| NLR≥1.83 | 0.690 | 0.0380 | 0.613-0.760 |
| Age≥45year | 0.589 | 0.0408 | 0.510-0.665 |
| Morphology irregular | 0.794 | 0.0321 | 0.724-0.853 |
| Microcalcification | 0.726 | 0.0356 | 0.651-0.792 |
| A/T ratio≥1 | 0.725 | 0.0298 | 0.650-0.792 |
| Tumor diamension≥10mm | 0.694 | 0.0382 | 0.617-0.763 |
| **External Validation cohort 2** |  |  |  |
| Nomogram | 0.833 | 0.0359 | 0.762-0.890 |
| NLR≥1.83 | 0.568 | 0.0441 | 0.483-0.651 |
| Age≥45year | 0.582 | 0.0434 | 0.497-0664 |
| Morphology irregular | 0.665 | 0.0244 | 0.581-0.742 |
| Microcalcification | 0.624 | 0.0427 | 0.540-0.704 |
| A/T ratio≥1 | 0.639 | 0.0258 | 0.554-0.717 |
| Tumor diamension≥10mm | 0.746 | 0.0296 | 0.666-0.815 |
|  |  |  |  |

NLR，Neutrophil-to-Lymphocyte Ratio；AUC，Area Under the Curve；SE，Standard Error；CI， Confidence

Interval ；A/T ratio≥1，anteroposterior-to-transverse diameter ratio
